# Relationships between the qNOX, qCON, burst suppression ratio, and muscle activity index of the CONOX monitor during total intravenous anesthesia: a pilot study

**DOI:** 10.1007/s10877-024-01214-6

**Published:** 2024-09-12

**Authors:** Federico Linassi, Sergio Vide, Ana Ferreira, Gerhard Schneider, Pedro Gambús, Matthias Kreuzer

**Affiliations:** 1 Department of Pharmaceutical and Pharmacological Sciences, University of Padova, Padova, Italy; 2 Department of Anesthesiology and Intensive Care, School of Medicine and Health, Technical University of Munich, Munich, Germany; 3 Department of Anesthesiology, Unidade Local de Saúde de São João, Porto, Portugal; 4 Systems Pharmacology Effect Control & Modeling (SPEC-M) Research Group, Anesthesiology Department, Hospital CLINIC de Barcelona, Barcelona, Spain; 5 RISE-Health, Medical Faculty of University of Porto, Porto, Portugal; 6 Faculdade de Engenharia, LAETA/INEGI, Universidade do Porto, Porto, Portugal; 7 NeuroImmunology Research Group, Institut d’Investigacions Biomèdiques August Pi i Sunyer (IDIBAPS), Barcelona, Spain

**Keywords:** Anaesthesia, General, Anaesthesiology, Electroencephalography, Patient monitoring, Propofol

## Abstract

Background: Processed electroencephalographic (EEG) indices can help to navigate general anesthesia. The CONOX (Fresenius Kabi) calculates two indices, the qCON (hypnotic level) and the qNOX (nociception). The CONOX also calculates indices for electromyographic (EMG) activity and EEG burst suppression (BSR). Because all EEG parameters seem to influence each other, our goal was a detailed description of parameter relationships. Methods: We used qCON, qNOX, EMG, and BSR information from 14 patients receiving propofol anesthesia. We described index relationships with linear models, heat maps, and box plot representations. We also evaluated associations between qCON/qNOX and propofol/remifentanil effect site concentrations (ceP/ceR). Results: qNOX and qCON (qCON = 0.79*qNOX + 5.8; *p* < 0.001; R^2^ = 0.84) had a strong linear association. We further confirmed the strong relationship between qCON/qNOX and BSR for qCON/qNOX < 25: qCON=-0.19*BSR + 25.6 (*p* < 0.001; R^2^ = 0.72); qNOX=-0.20*BSR + 26.2 (*p* < 0.001; R^2^ = 0.72). The relationship between qCON and EMG was strong at higher indices: qCON = 0.55*EMG + 33.0 (*p* < 0.001; R^2^ = 0.68). There was no qCON > 80 without EMG > 0. The relationship between ceP and qCON was qCON=-3.8*ceP + 70.6 (*p* < 0.001; R^2^ = 0.11). The heat maps also suggest that the qCON and qNOX can at least partially separate the hypnotic and analgetic components of anesthesia. Conclusion: We could describe relationships between qCON, qNOX, EMG, BSR, ceP, and ceR, which may help the anaesthesiologist better interpret the information provided. One major finding is the dependence of qCON > 80 on EMG activity. This may limit the possibility of detecting wakefulness in the absence of EMG. Further, qNOX seems generally higher than qCON, but high opioid doses may lead to higher qCON than qNOX indices.

**Supplementary Information:**

The online version contains supplementary material available at 10.1007/s10877-024-01214-6.

## Introduction

The CONOX (Fresenius-Kabi AG, Bad Homburg, Germany) is a patient monitoring system that calculates two indices, the qCON and qNOX, from the electroencephalogram (EEG) of a patient and also presents information regarding electromyographic activity (EMG) as well as the burst suppression ratio (BSR). The qNOX and qCON are designed to reflect the hypnotic level of anesthesia, given by the index qCON, as well as to measure nociception (in particular, the probability of movement in response to painful stimulation), provided by the index qNOX [1]. The qCON and qNOX are derived by combining the information of frontal EEG power in different frequency bands. The performed combinations are proprietary and initially based on an Adaptive Neuro Fuzzy Inference System (ANFIS) [1]. Both indices range from 0 to 99. According to the manufacturer’s recommendation, high values of the qCON indicate a lighter level of anesthesia/wakefulness, and the range between 40 and 60 seems appropriate for surgical intervention. For the qNOX, the 40 to 60 index range indicates a low probability for a movement response to a surgical stimulus. Higher qNOX indices (i.e., qNOX > 60) reflect a higher, whereas lower indices (qNOX < 40) indicate a lower probability of such a reaction. In addition to the qNOX and qCON, the CONOX provides the BSR that helps to detect excessively deep levels of anesthesia as well as an EMG index that reflects muscle activity. In the case of a non-zero BSR, the patient may be in an excessively deep anesthetic level with waxing and waning EEG patterns [2]. The introductory article for the qCON/qNOX describes a strong relationship between BSR, qCON, and qNOX for qCON < 25 [1]. The EMG index indicates the possibility of muscle activity influencing the EEG. The EMG information seems to play a crucial role in the processed EEG monitors’ approach to detecting wakefulness, at least for the BIS [3, 4]. In general, all these processed EEG parameters of a monitor seem to be interwoven and the better we know how these indices relate to each other, the better we can deal with situations where the provided information may be contradictory. Therefore, we conducted analyses that helped to understand the behavior of the CONOX indices in more detail.

## Methods

### Patients

For our retrospective analyses, we included data from 15 patients, after obtaining Institutional Review Board and Ethics in Clinical Research Committee approval (Hospital CLINIC de Barcelona nº HCB/2016/0318v2). Each patient gave written consent to participate in the study. Patients were scheduled for ambulatory gynecologic procedures and general surgery (surgical hysteroscopy, laparoscopy, and urinary incontinence correction). We recruited these patients in the Ambulatory Surgery Unit at the Hospital CLINIC (Barcelona, Spain) during the month of July 2018. Exclusion criteria included prior eye surgery, any ocular diseases besides refraction errors (because the study included pupillary measurements), prescription of drugs affecting the size or reflex of the pupil, and morbid obesity (BMI > 35). Because this is a retrospective investigation, we did not conduct a power analysis.

### Study protocol

Upon arrival to the operating room, we attached the patient to routine monitors including a continuous electrocardiogram, pulse oximetry, and non-invasive blood pressure. We did not administer any premedication. We used total intravenous anesthesia with propofol and remifentanil for induction and maintenance of general anesthesia using a Target Controlled Infusion (TCI) system (Base Primea docking station, Fresenius Kabi AG, Bad Homburg, Germany).

We induced loss of consciousness by targeting the effect-site concentration of propofol (CeP, Schnider model) between 5 and 11 µg·mL^− 1^ [5, 6]. Further, we defined the range for the effect site concentration of remifentanil (CeR, Minto model) from between 0.5 and 6 ng mL^− 1^ [7, 8]. varying between 0.5 and 6 ng mL^− 1^. The applied criss-cross approach enabled enough combinations of propofol and remifentanil administration to cover the whole surface of clinically relevant concentrations [9]. After two minutes of pseudo-equilibration we secured the airway either by placement of a laryngeal mask or by endotracheal intubation. In the cases requiring tracheal intubation, we administered 30 mg of rocuronium bromide two minutes before laryngoscopy.

During anesthesia maintenance, we titrated the hypnotic level using the CONOX, while the analgesic effect was titrated according to the anaesthesiologist’s discretion. Because of our limited sample size, we refrained from conducting a subgroup analysis regarding the effect of the neuromuscular blocking agent on the EEG.

We recorded data from the CONOX (qCON, qNOX, BSR, EMG, and raw EEG) in real time using the CONOX view (Fresenius Kabi, Bad-Homburg, Germany). The EEG electrodes were placed on the patients’ forehead according to the manufacturers’ specifications. CeP (µg mL-1) and CeR (ng mL-1) were recorded using the Rugloop software (DEMED, Gent, Belgium).

### Analysis of the trend data

For our analyses, we first cleaned the data set from invalid index values and synced all indices provided by the CONOX each second with the effect site concentrations of remifentanil and propofol provided every 2 s. To avoid reducing the sample size, we did not separate the perioperative period into different episodes. Further, with this pilot investigation, we were interested in the overall associations of the indices.

To evaluate the behavior of the indices we investigated the following relationships:

1. Between qCON and EMG to understand the influence of muscle activity on the generation of qCON indicating wakefulness. This is important information for the possible detection of intraoperative awareness.
2. Between qCON and BSR to understand the qCON index range where burst suppression can be expected.
3. Between qCON or qNOX and the effect site concentrations of propofol and the opioid.
4. Between qCON and qNOX to understand possible relationships between these indices reflecting the hypnotic and analgetic component of general anesthesia.

We used both the pooled data, i.e., each valid index combination from all patients (all valid data collected), as well as median values per patient for our analyses and figures. Therefore, we derived the median value of a parameter like EMG or qNOX for each qCON value observed. This means, for example, that patient 1 had a median EMG of 80 when having a qCON of 90.

### Statistics

To properly present and describe our results, we applied descriptive as well as inference statistical approaches. We describe the overall relationships among the indices using the pooled data, i.e., the valid index pairs from all patients combined. We are aware that the data are dependent and that patients with a higher number of recorded data pairs are overrepresented. Hence, we also calculated median values per patient, e.g., the median qNOX at a defined qCON index to generate the box and scatter plots. We also present violin plots that were created with the *violin* function by H. Hoffman, available at mathworks.com. We built a linear model using the MATLAB *fitlm* function inclusive t-statistics and coefficient of determination (R2). For the investigation of the qNOX or qCON being higher, we used the AUC parameter from the MES toolbox [10] together with 10k-fold bootstrapped 95% confidence intervals (Ci). A 95%Ci that excludes 0.5 indicates an effect difference from chance, i.e., a significant difference in our analyses. We used MATLAB R2017b (The Mathworks, Natick, MA, USA) for statistical analysis and the graphical representation of the results.

## Results

From the 15 patients, we derived a total of 68,125 qCON, qNOX, EMG, and BSR pairs, i.e., 68,125 s (∼ 19 h), to use for our analyses. We had to exclude a number of points because of invalid values, dependent on the comparisons. The supplemental Table S1 contains the number of included pairs or triplets for the analyses This included one patient with an entire invalid recording due to EEG recording issues.

The recordings from the remaining 14 patients lasted between 2315 and 13,192 s (median: 4450 s) and the proportion of valid data for the individual patient ranged from 86 to 100% with a median of 99%.

### Demographics

Of the 14 patients included in the study, 11 were female and 3 were male. The median age was 40.5 years (1st-3rd quartile: 35-70.25) years, and the median Body Mass Index was 25.1 (22.2–27.9).

### Relations between qCON or qNOX and EMG

**Fig. 1:**
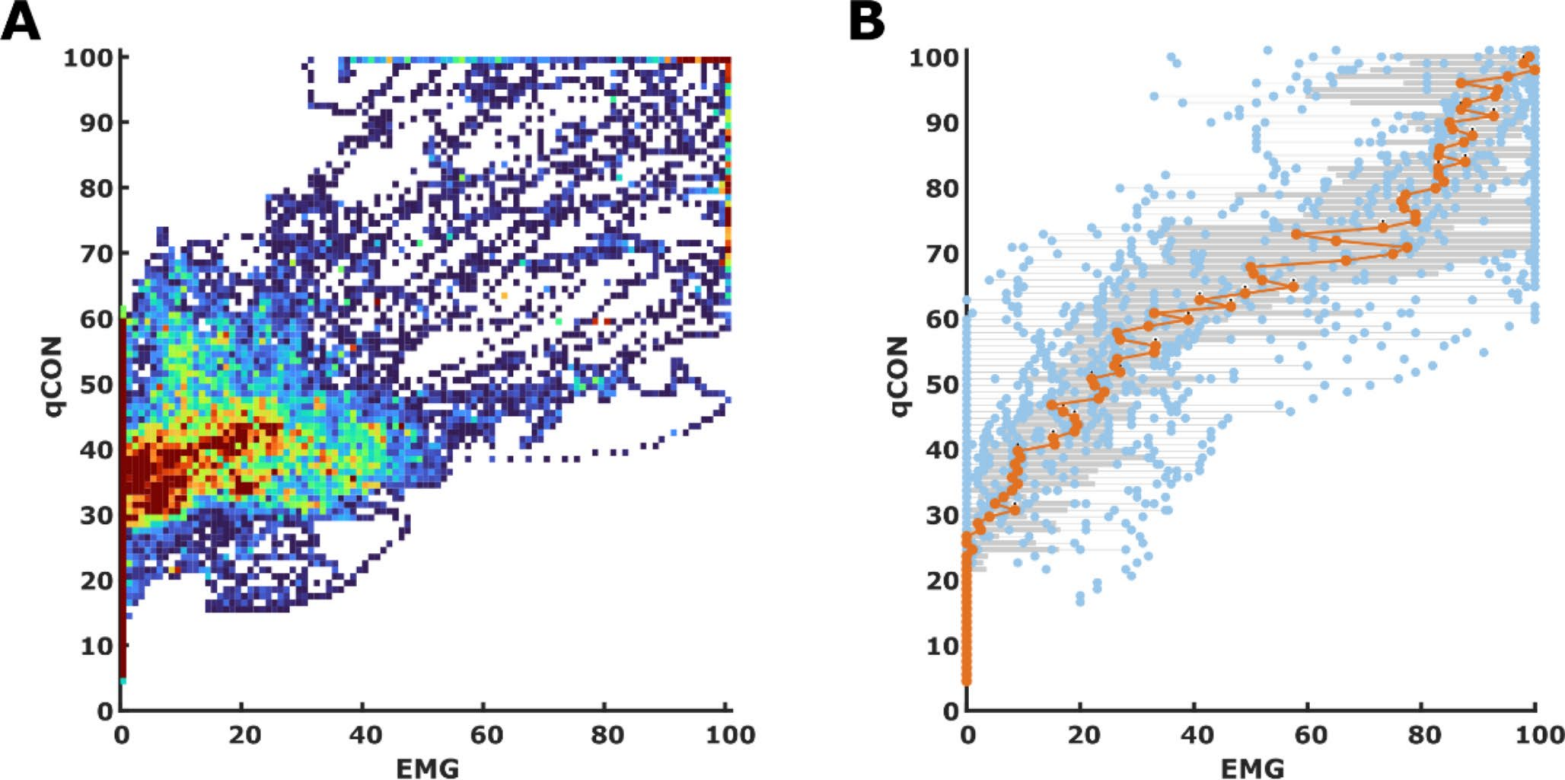
Heat map and box plot for the qCON to EMG relationship. **A)** The heat map presents the distribution of qCON / EMG pairs for all data pairs recorded. **B)** The box and scatter plot were derived from the median EMG for each observed qCON in the single patients (*n* = 14). The grey boxes indicate the 25th and 75th percentile with whiskers spanning to the most extreme values that are not considered outliers. The blue dots present the single median EMG values, and the orange line and dots indicate the median EMG value for each qCON value. The most important finding is the observation that qCON values above 80, indicative of an awake patient, only occur with EMG > 0

The evaluation of the relationship between the qCON or qNOX and the calculated EMG index revealed a strong dependence of qCON > 80 and EMG in our data set. A qCON > 80 only occurred in the presence of substantial EMG activity. EMG activity was also indicated for lower qCON but the EMG indices could span the entire scale for these qCON indices. The detailed heat map and box plot are presented in Fig. 1. The linear model derived from the median EMG values per qCON index for each patient was: qCON = 0.55*EMG + 33.0 (*p* < 0.001; t-Stat = 47.38; R^2^ = 0.68). For the qNOX vs. EMG investigation we observed high qNOX values without EMG. The linear model derived from the median EMG values per qNOX index for each patient was: qNOX = 0.59*EMG + 40.3 (*p* < 0.001; t-Stat = 35; R^2^ = 0.53). We present the corresponding plots as supplemental Figure S1.

### Relations between qCON or qNOX and BSR

**Fig. 2:**
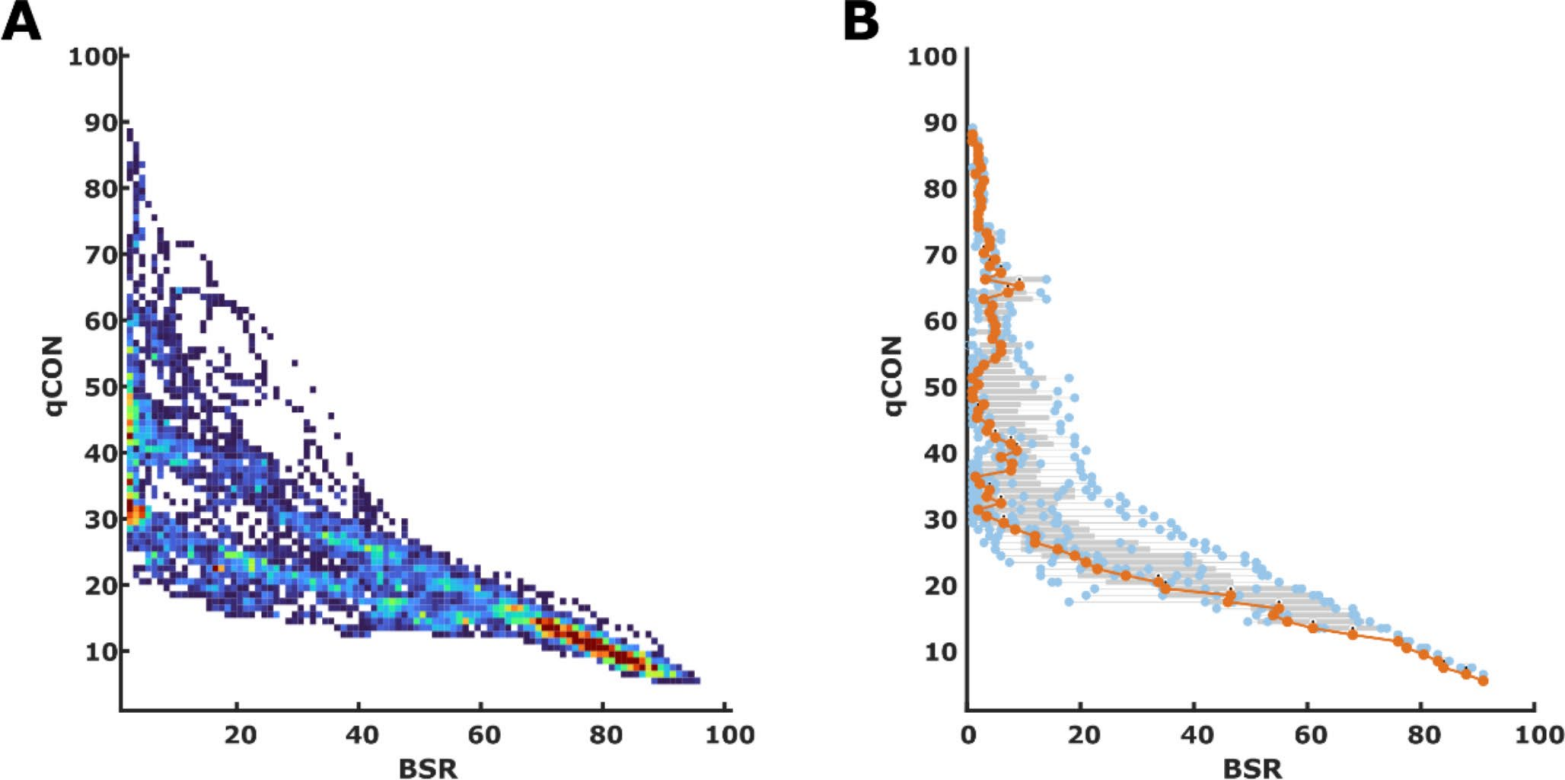
Heat map and box plot for the qCON to burst suppression ratio (BSR) relationship. **A)** The heat map presents the distribution of qCON / BSR pairs for all data pairs recorded. **B)** The box and scatter plot were derived from the median BSR for each observed qCON in the single patients (*n* = 10). The grey boxes indicate the 25th and 75th percentile with whiskers spanning to the most extreme values that are not considered outliers. The blue dots present the single median BSR values, and the orange line and dots indicate the median BSR value for each qCON value

We observed positive BSR in 10 patients. For these analyses, we only considered data with BSR > 0. Figure 2 presents the heat map plots of the observed qCON/BSR pairs. The linear model derived from the median BSR values per qCON index for each patient was: qCON=-0.57*BSR + 46.8 (*p* < 0.001; t-Stat=-16.0; R^2^ = 0.43). When only considering qCON < 25, i.e., when the BSR is supposed to take over, the linear model was qCON=-0.19*BSR + 25.6 (*p* < 0.001; t-Stat=-17.4; R^2^ = 0.72). We found similar results for the qNOX with qNOX=-0.67*BSR + 53.4 (*p* < 0.001; t-Stat=-16.8; R^2^ = 0.41) for the regression of qNOX with BSR > 0 and qNOX=-0.19*BSR + 26.2 (*p* < 0.001; t-Stat=-16.1; R^2^ = 0.71) for all qNOX < 25. **Figure S2** contains the heat map and the box plot. For both indices qCON and qNOX, the boxplots indicate that BSR > 0 with a qCON or qNOX > 30 is a spurious observation.

### Relations between qCON and qNOX

**Fig. 3:**
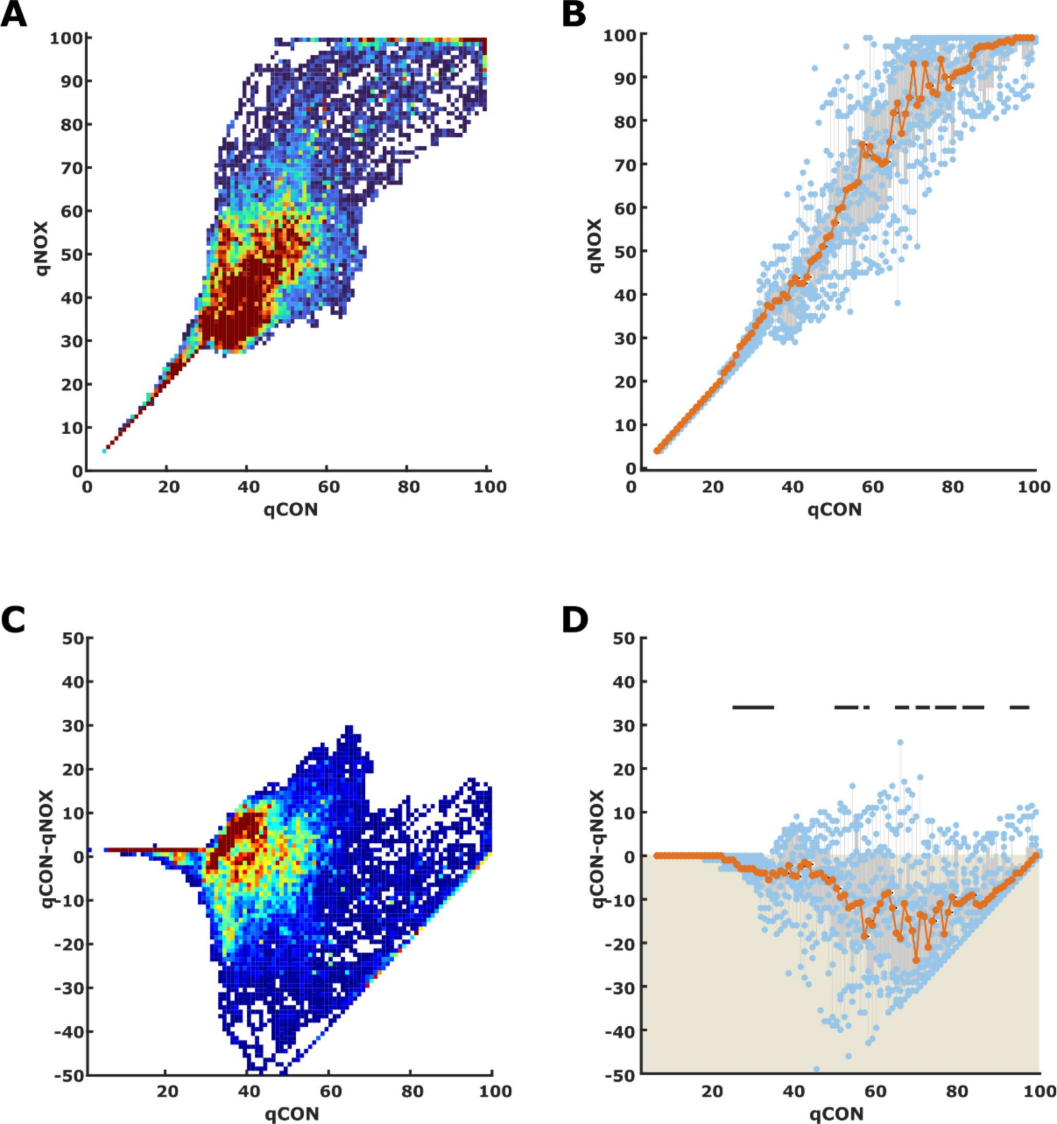
Heat map and box plots for the qCON to qNOX relationships. **A)** The heat map presents the distribution of qCON / qNOX pairs for all data pairs recorded. **B)** The box and scatter plot were derived from the median qNOX for each observed qCON in the single patients (*n* = 14). **C)** The heat map presents the distribution of qCON / qCON-qNOX pairs for all data pairs recorded. **D)** The box and scatter plot were derived from the median qCON-qNOX for each observed qCON in the single patients (*n* = 14). The grey boxes indicate the 25^th^ and 75^th^ percentile with whiskers spanning to the most extreme values that are not considered outliers. The blue dots present the single median BSR values and the orange line and dots indicate the median qNOX value for each qCON value

We found a strong relationship between qCON and qNOX as displayed in Fig. 3. The linear model was qCON = 0.79*qNOX + 5.8 (*p* < 0.001; t-Stat = 75.1; R^2^ = 0.84). The heat map in Fig. 3A resembles a diving kingfisher bird. The beak presents the linear relationship between qCON and qNOX at low indices: For qCON indices up to 25 and qNOX indices up to 28 there seems to be an almost linear relationship between both parameters. In 29% of cases, the qCON was higher than the qNOX, and in 50% of the cases, it was the other way around, as depicted in Fig. 3C. The box plot in Fig. 3D shows that the qNOX seems generally higher than the qCON, and the AUC analysis revealed a significantly higher qNOX than the qCON at qCON indices, indicating deep, adequate, or light anesthesia as well as sedation. When the qCON indicated a fully awake patient, the qNOX was not lower than 80, but the qCON range was wider, ranging from ∼ 45 to 99 at maximum qNOX indices.

### Relationships between indices and effect site concentrations

**Fig. 4:**
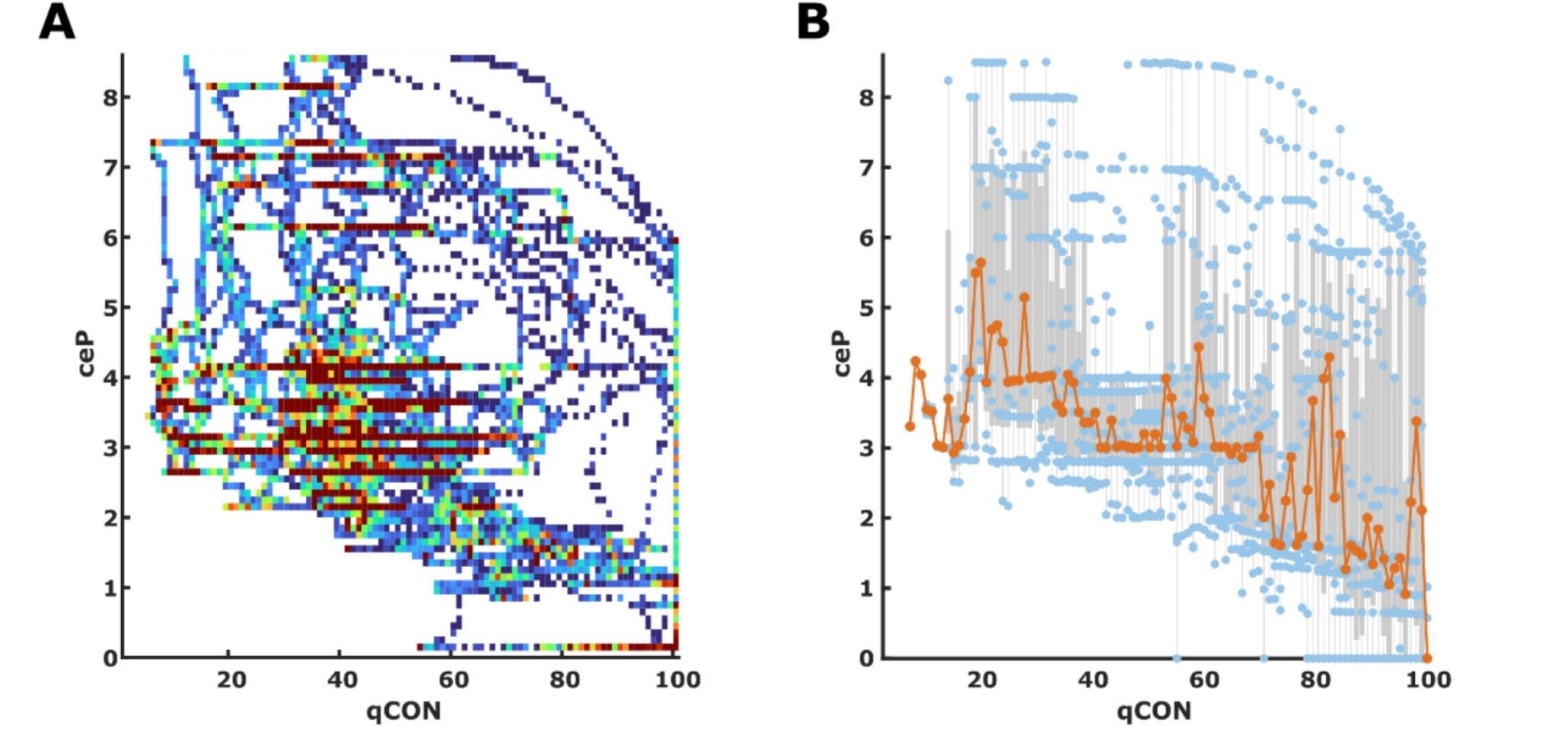
Heat map and box plot for the qCON to propofol effect-site concentration (ceP) relationship. **A)** The heat map presents the distribution of the qCON / ceP pairs for all recorded data pairs. **B)** The box and scatter plot were derived from the median ceP for each observed qCON in the single patients (*n* = 14). The grey boxes indicate the 25th and 75th percentile, with whiskers spanning to the most extreme values that are not considered outliers. The blue dots present the single median BSR values, and the orange line and dots indicate the median ceP value for each qCON value

We observed the expected decrease of qCON with ceP: qCON=-3.8*ceP + 70.6 (*p* < 0.001; t-Stat=-11.5; R^2^ = 0.11). The rather low coefficient of determination R^2^ indicates that only little variability can be explained by the linear regression model. In 9.4% of the times when ceP was higher than 5 µg·mL^− 1^, we observed a qCON > 60, i.e., a stated level of light anesthesia, sedation, or awake. Figure 4 presents the qCON/ceP heatmap and box plots. The box plot in supplemental Figure S3 describes the occurrence of burst suppression by means of the BSR that started at a median ceP of 3 µg·mL^− 1^ or more. We also found a significant linear trend between ceR and qNOX with qNOX=-10.7*ceR + 74.5 (*p* < 0.001; t-stat=-21.0; R^2^ = 0.28). The corresponding heatmap and boxplot can be found in supplemental Figure S4. But of course there is a significant interaction effect between ceP and ceR influencing the qNOX (*p* = 0.012; t-stat = 2.51). Supplemental Figure S5 shows the relationships between the differences of the two indices and the effect site concentrations of propofol and remifentanil. We found that in general, the qCON was higher than the qNOX at lower propofol concentrations and higher remifentanil concentrations.

## Discussion

We can describe the relationship between the CONOX indices presented in a more detailed fashion than previously reported. Main findings are the dependence of high qCON values on positive EMG, a strong general correlation between qCON and qNOX, generally higher qNOX than qCON values, and the confirmation of the linear relationship between BSR and qCON or qNOX.

### Monitoring the hypnotic and analgesic component

The CONOX monitor claims to combine monitoring the hypnotic and analgesic components of anesthesia. The qCON is designed to evaluate the anesthetic level and the qNOX to present the patient’s probability to react to a noxious stimulus. In an initial study, Melia et al. [11]. described different reactions of these indices to different stimuli. Hence, the CONOX seems to be the only EEG-based monitor that combines hypnosis and nociception monitoring besides the brain anesthesia response monitor (Cortical Dynamics, Scoresby, Victoria, Australia) [12]. 

Monitoring the hypnotic component of anesthesia is based on the evaluation of the anesthetic-induced change from a fast and low-amplitude EEG during wakefulness to a slow and high-amplitude EEG during general anesthesia for common anesthetics [13]. Monitoring the analgesic component is more complex since different EEG reactions to a noxious stimulus have been described. An increase in EEG beta power can be observed during intubation or incision and is often accompanied by movement [14, 15]. A loss of dominant alpha-band activity can occur as a reaction to a noxious stimulation intraoperatively, [16, 17] and ‘paradoxical’ increases in delta power following the stimulation were described as well [18]. The qNOX mainly seems to react to beta arousal [1]. A recent study describes the absence of correlation between maximum qNOX values at the end of surgery and postoperative pain [19], which is not the task of the qNOX being designed as a measure of probability of movement reaction to noxious stimulation [1]. One major point of criticism of this study was the absence of other indices like the qCON presented [20]. In the Ledowski study, the qNOX indices covered a wide range from around 30 to over 80 [19]. Because qCON and qNOX seem correlated, the investigated patients probably were at different anesthetic levels. Our results highlight the necessity to evaluate both indices together. While we cannot answer data regarding the qNOX performance for the other EEG reactions to a noxious stimulus, we can describe the relationship between the qCON and the qNOX in detail. Because we observed cases of higher qCON than qNOX indices at high opioid concentrations and higher qCON than qNOX indices at high propofol concentrations, we very cautiously state that qCON and qNOX may indeed look at two different components of general anesthesia, but with a big overlap and strong interaction terms. This is in line with current findings, [11, 21] but they did not include interactions. However, our study was conducted only with patients receiving propofol and remifentanil. Hence, future studies are necessary to investigate the qCON and qNOX relationship for different general anesthetics (i.e., inhalational ones). Hence, the correlation between qNOX and qCON may be different (lower) for certain regimens. To investigate the analgesic component in a proper fashion, the indices could be derived from awake subjects receiving an opioid only.

### Relationships between the indices

Not a lot of literature is available regarding the qCON and qNOX relationship. Regarding state transitions, the qCON seems to decrease faster during anesthesia induction, whereas the qNOX increases earlier during emergence and reacts faster to the laryngeal mask insertion stimulus [11]. The different delays may cause contradicting qCON and qNOX indices. Further, we could confirm the almost perfect linear relationship of BSR and qCON or qNOX previously described [1]. This strong dependence seems very similar to the bispectral index to BSR relationship [22]. 

We can describe strong overall relationships between qCON, qNOX, and BSR. Especially in situations with detected burst suppression the linear relationship between qCON and qNOX is nearly perfect. But we also observed situations where high qCON and qNOX values occurred in combination with BSR > 0. These contradicting indices can most probably be attributed to the different time delays of index calculation [23]. We also observed high qCON values at high propofol concentrations > 5 µg/ml that cause burst suppression with high probability [24, 25]. This may be indicative of burst suppression not detected by the monitor. Other monitoring systems seem to underestimate the occurrence of burst suppression [26] which then can cause high index values [27]. But further studies are necessary to evaluate if this applies to the CONOX. Further, the time delay of index calculation [23] may also cause situations with high qCON values at high ceP. For the levels of anesthesia without burst suppression, the qNOX seemed to have a tendency to be higher than the qCON. This makes sense because, at least for volatile anesthetics, the blockage of cardiovascular response to surgical incision occurs at 1.3 minimum alveolar concentration (MAC), i.e., at a later point in the course of anesthesia. Hence, qNOX may remain higher. With opioids, the 1.3 MAC threshold can be reduced to below 1 MAC[28] which should influence the difference between qCON and qNOX. We did not find any qNOX < 70 and qCON ≥ 80, indicating an awake patient. This observation confirms the assumption that an awake patient has a high probability of responding to painful stimulation. Generally, the qNOX was higher than qCON. But at low propofol and high remifentanil concentrations, we found qCON > qNOX. This may point towards the strength of each index to either reflect the hypnotic or analgesic component of anesthesia.

A major finding of our investigation presents the dependence of high qCON values on EMG activity in our data set. An influence of EMG on high qCON values was suggested earlier [1]. We did not observe qCON indices reflecting sedation or wakefulness with an EMG index below 20. Hence, our results could indicate that neuromuscular activity seems crucial for the qCON to indicate wakefulness, but this finding has to be confirmed in the future. Studies with the BIS showed a causal relationship between EMG activity and high BIS values, as the BIS decreased in awake volunteers with a neuromuscular blockade [3, 4]. The CONOX EMG index is derived from the frontal EEG that also is used for qCON and qNOX calculation. Because, in general, the frequency ranges of the recorded EEG and EMG signal overlap [29] and arousal reactions and wakefulness are normally accompanied by EMG activity, assessment of the “EEG only” during these episodes is almost impossible. This most probably is the cause for our observations of high qCON values only in combination with EEG activity. If the qCON is capable of reliably detecting (intraoperative) wakefulness during neuromuscular blockade is subject to further investigations. To completely answer this question, data from subjects that are awake during a neuromuscular blockade would be necessary. In a study by Schuller et al., volunteers were administered solely neuromuscular blocking agents [3]. Using the isolated forearm technique, they demonstrated that while these volunteers were awake, their BIS values could be below 60 for significant periods of time. This emphasized the importance of EMG for BIS, and demonstrated that values deemed adequate for surgical anesthesia were obtained for awake patients. Bearing this in mind, and based on our findings, the clinician may also need to be aware of situations where the qCON is used in patients undergoing neuromuscular blockade.

The qNOX, in contrast, does not seem to depend as much on the EMG, but Jensen et al. described a qCON as reaction to noxious stimulation with EMG activation [1]. Future studies should relate qCON and qNOX values to other, non-EEG based techniques of detecting consciousness during general anaesthesia with neuromuscular blockade like for instance the isolated forearm technique as has been done in a recent meta-analysis [30]. 

### Limitations

We only present results for propofol cases. We did not record anaesthetic and surgical events (i.e. start of induction, intubation, skin incision), so we cannot present separate plots for each of these moments. Future studies should also compare qCON and qNOX to other non-EEG based analgesia. Further, as mentioned in the methods, we did not evaluate the impact of neuromuscular blocking agents on the indices. This should be investigated in the future with a sufficiently large data set.

### Clinical impact

Our results can help to better understand the CONOX monitor. The dependence of qCON on EMG and the knowledge that other indices, i.e., the BIS may not be able to detect an awake patient without EMG [3] could tell the anesthesiologist always to consult the displayed raw EEG as well. At some point, the qNOX and the qCON are driven by the BSR. In case the monitor does not detect burst suppression -other devices like the SEDLine seem to underestimate the real occurrence of burst suppression [26]- the qCON and qNOX may be implausible and again, the raw EEG should be consulted. Our pilot results regarding a potential separation of the hypnotic (qCON) and analgesic component (qNOX) could help the anesthesiologist to improve the quality of anesthesia by including both indices in decision-making.

## Conclusion

We could show a dependence of high qCON indices on EMG activity, a generally higher qNOX than qCON during levels of general anesthesia, as well as strong correlation between low qNOX and qCON values and BSR. We could also describe relationships between qCON and qNOX and the modelled effect site concentrations of propofol and opioids.

## Electronic supplementary material

Below is the link to the electronic supplementary material.

Supplementary Material 1

## Data Availability

Data can be made available upon request.

## Code availability

The codes may be available upon request
