# Supplementary material for: Relationships between the qNOX, qCON, burst suppression ratio, and muscle activity index of the CONOX monitor during total intravenous anesthesia: a pilot study
Source: J Clin Monit Comput. 2024 Sep 12;38(6):1281–90. doi: 10.1007/s10877-024-01214-6 (PMC11604674; doi:10.1007/s10877-024-01214-6)
Supplement: Supplementary file 1 — Supplementary Material 1 [file 10877_2024_1214_MOESM1_ESM.docx]

**Supplemental information for: Relationships between the qNOX, qCON, burst suppression ratio, and muscle activity index of the CONOX monitor**


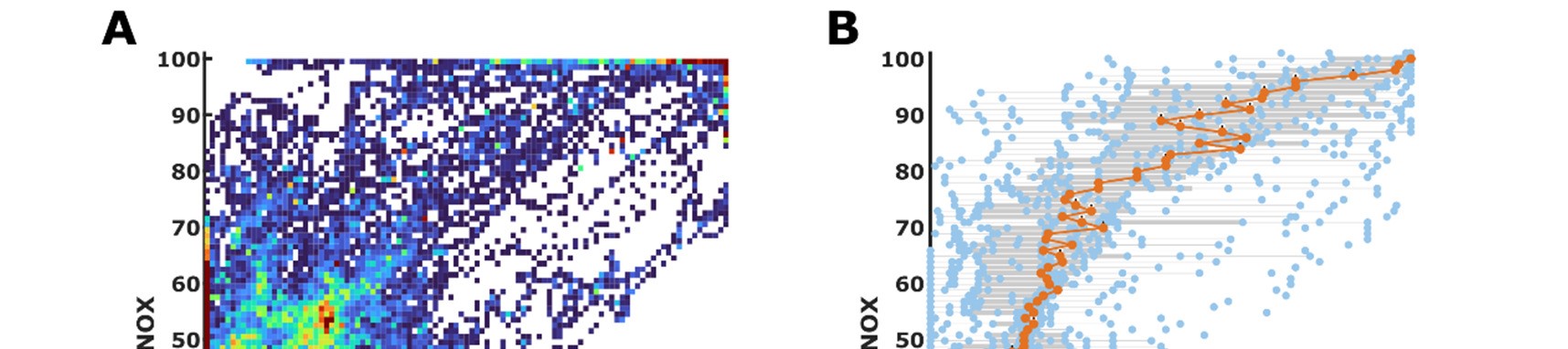

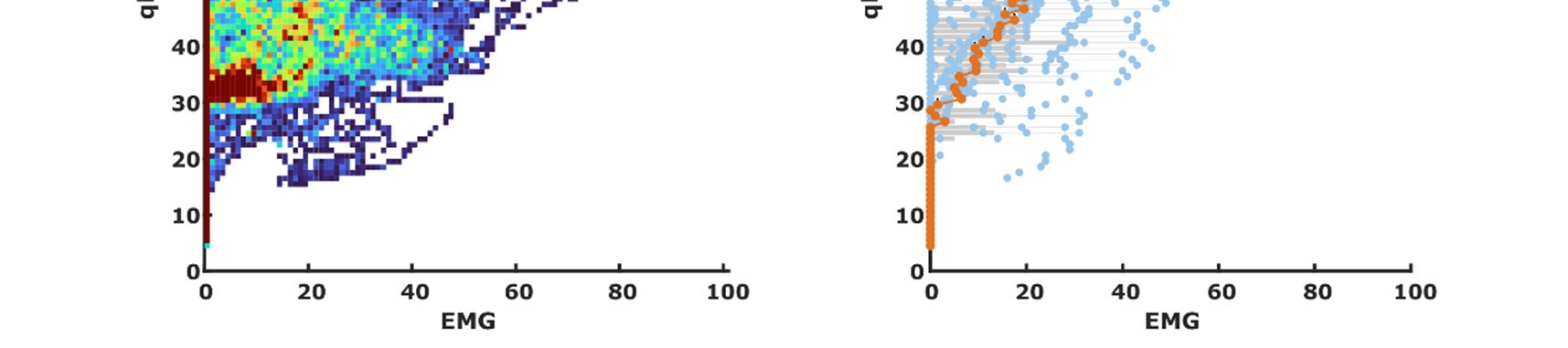


**Figure S1:** Heat map and box plot for the qNOX to EMG relationship

1. The heat map presents the distribution of qNOX / EMG pairs for all data pairs recorded.
2. The box and scatter plot was derived from the median EMG for each observed qNOX in the single patients (n=14). The grey boxes indicate the 25^th^ and 75^th^ percentile with whiskers spanning to the most extreme values that are not considered an outlier. The blue dots present the single median EMG values and the orange line and dots indicate the median EMG value for each qNOX value.

The most important finding is the observation that qNOX values above 80, indicative of an awake patient, only occur with EMG > 0. EMG: electromyogram


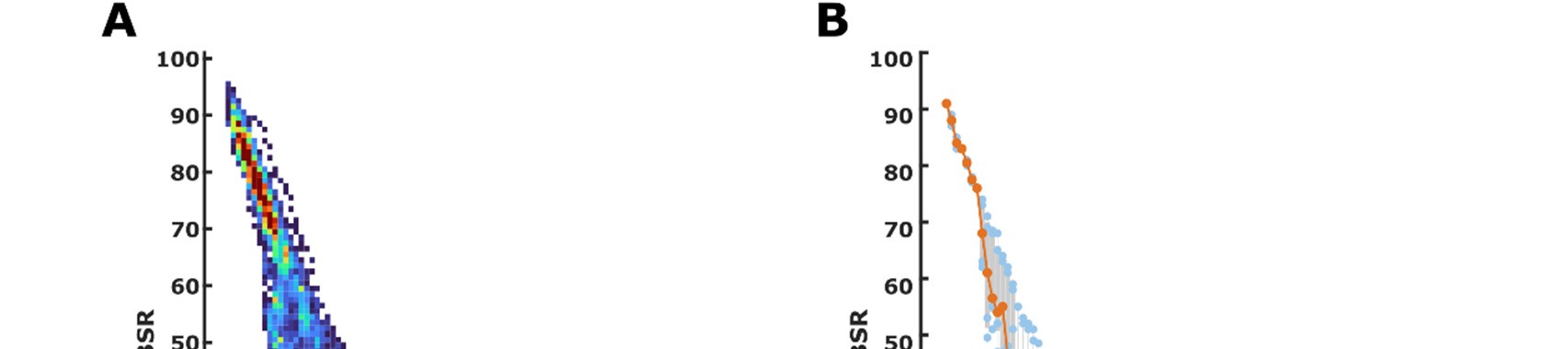

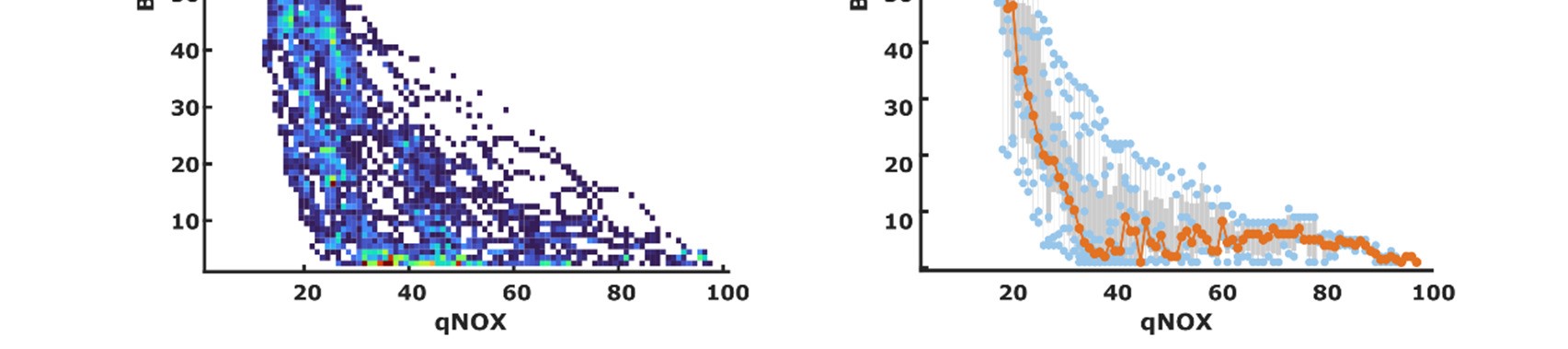


**Figure 2:** Heat map and box plot for the qNOX to burst suppression ratio (BSR) relationship

1. The heat map presents the distribution of qNOX / BSR pairs for all data pairs recorded.
2. The box and scatter plot was derived from the median BSR for each observed qNOX in the single patients (n=10). The grey boxes indicate the 25^th^ and 75^th^ percentile with whiskers spanning to the most extreme values that are not considered an outlier. The blue dots present the single median BSR values and the orange line and dots indicate the median BSR value for each qNOX value.


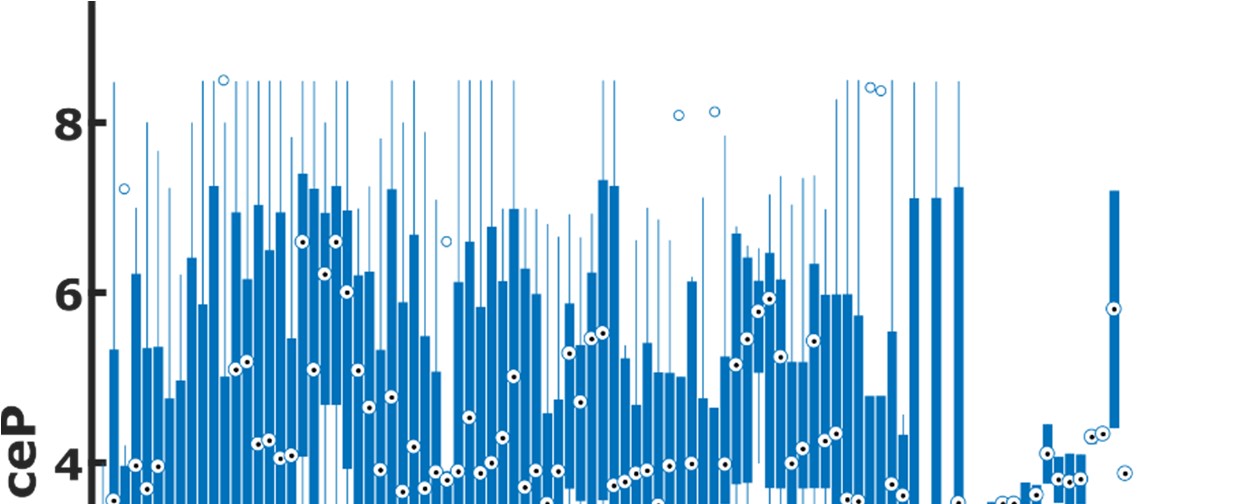

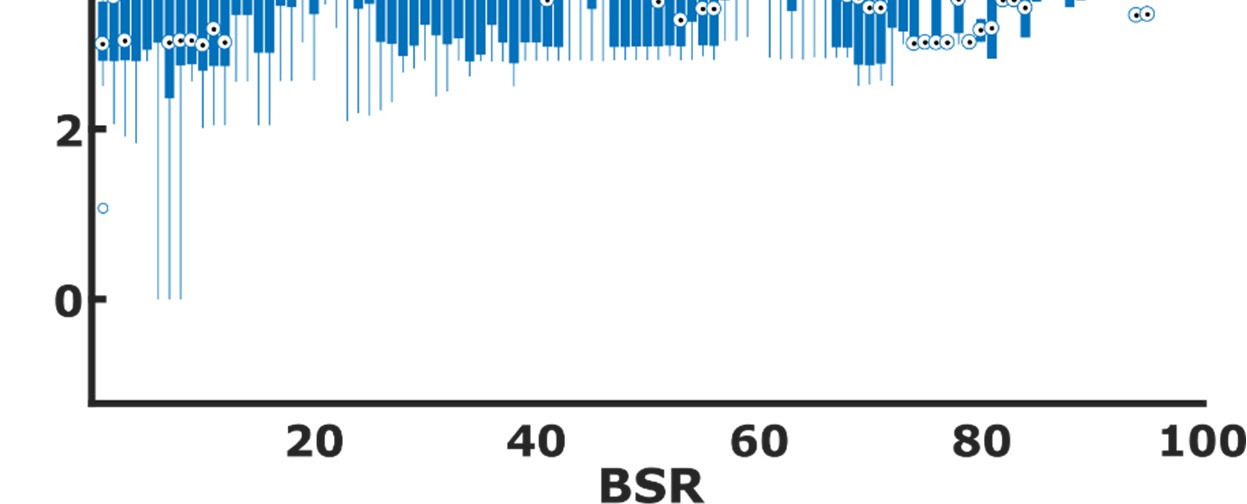


**Figure S3**: Boxplots of the ceP range for each BSR index. We observed burst suppression, i.e., a BSR>0 starting at median ceP of 3 µg·mL-1 or more.


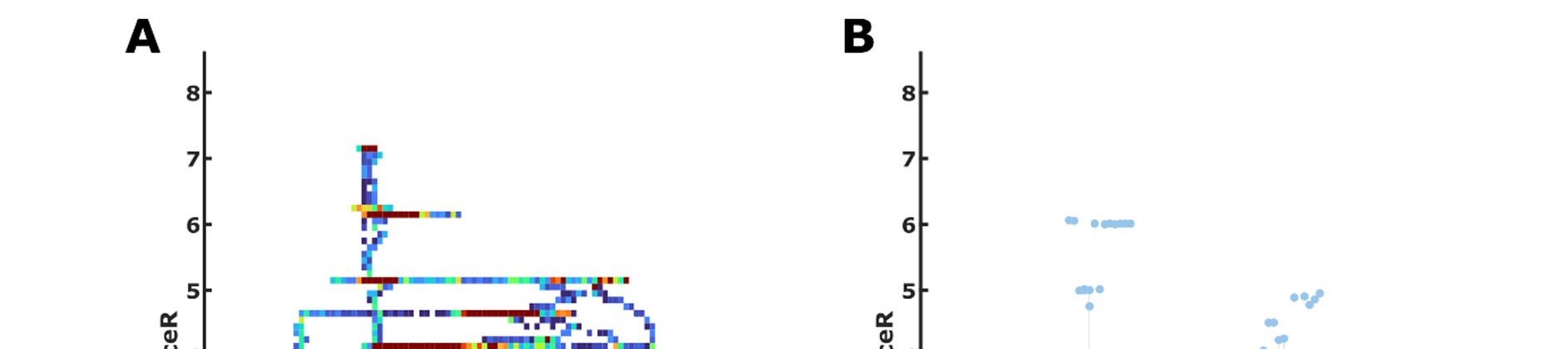

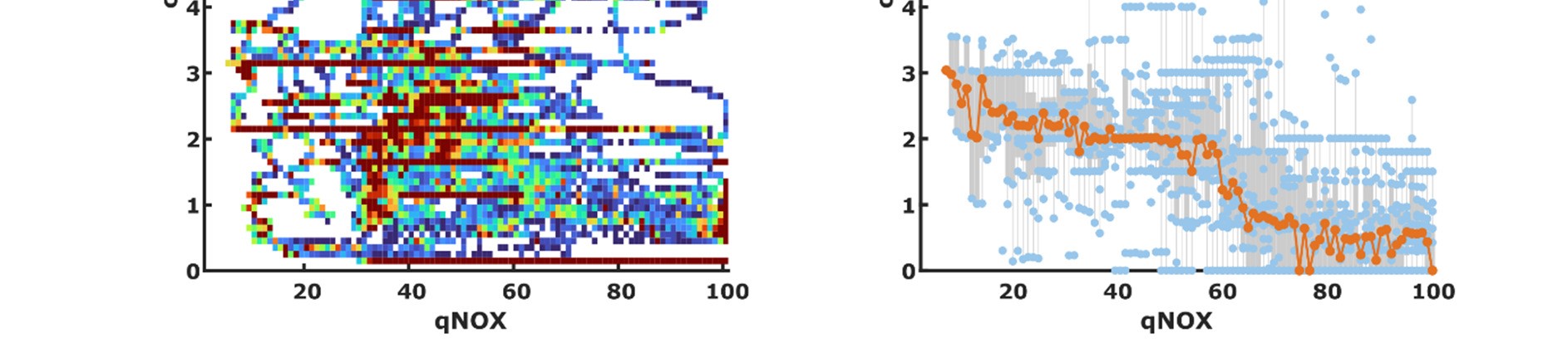


**Figure S4:** Heat map and box plot for the qNOX to remifentanill effect site concentration (ceR) relationship

1. The heat map presents the distribution of qNOX / ceR pairs for all data pairs recorded.
2. The box and scatter plot was derived from the median ceR for each observed qNOX in the single patients (n=14). The grey boxes indicate the 25^th^ and 75^th^ percentile with whiskers spanning to the most extreme values that are not considered an outlier. The blue dots present the single median BSR values and the orange line and dots indicate the median ceR value for each qNOX value.


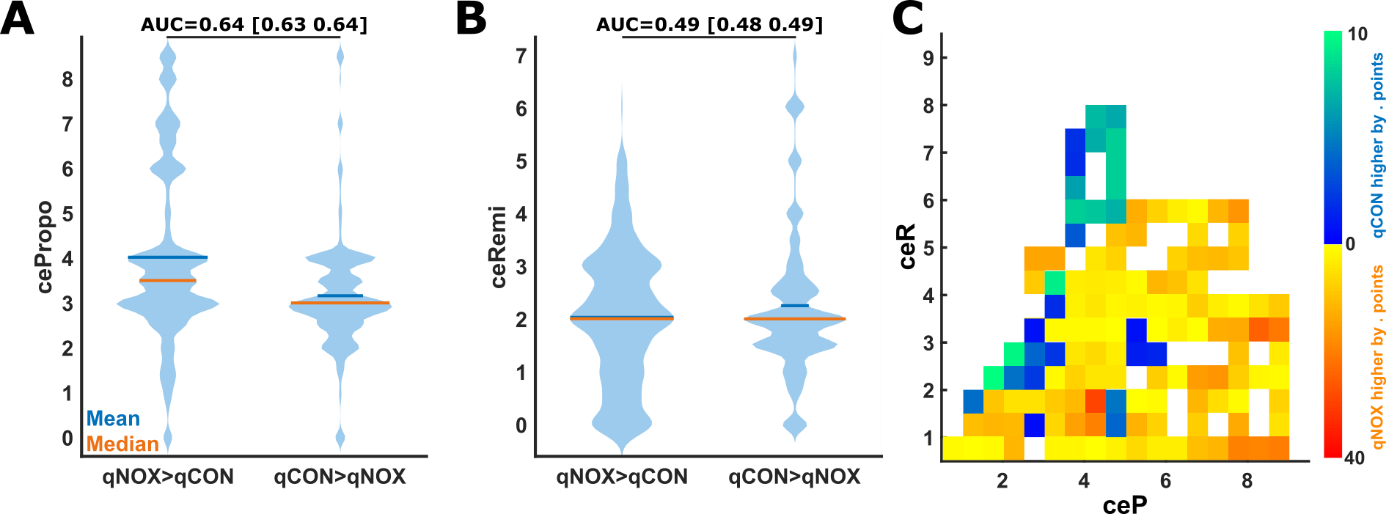


Figure S5: Relationships between the qNOX/qCON difference and the propofol and remifentanil effect site concentrations.

1. The qNOX is higher than the qCON at higher propofol concentrations.
2. There is no difference between the remifentanil concentrations for qCON>qNOX and qNOX>qCON
3. Median qCON-qNOX values for the different combinations of propofol and remifentanil effect site concentration. For most combinations, qNOX was higher qCON as indicated by the warm colors. Especially higher ceR and lower ceP, we observed qCON>qNOX as indicated by the cold colors.
